# Exploring the Potential of Yellow Mealworm (*Tenebrio molitor*) Oil as a Nutraceutical Ingredient

**DOI:** 10.3390/foods13233867

**Published:** 2024-11-29

**Authors:** Montserrat Martínez-Pineda, Teresa Juan, Agata Antoniewska-Krzeska, Antonio Vercet, María Abenoza, Cristina Yagüe-Ruiz, Jarosława Rutkowska

**Affiliations:** 1 Instituto Agroalimentario de Aragón—IA2, Universidad de Zaragoza—Centro de Investigación y Tecnología Agroalimentaria de Aragón (CITA), 50013 Zaragoza, Spain; tjuan@cita-aragon.es (T.J.); mabenoza@unizar.es (M.A.); cyague@unizar.es (C.Y.-R.); 2 Faculty of Health and Sports Science, Universidad de Zaragoza, Plaza Universidad 3, 22002 Huesca, Spain; vercet@unizar.es; 3 Centro de Investigación y Tecnología Agroalimentaria de Aragón (CITA), Av. de Montañana, 930, 50059 Zaragoza, Spain; 4 Institute of Human Nutrition Sciences, Faculty of Human Nutrition, Warsaw University of Life Sciences, Nowoursynowska St. 159c, 02-776 Warsaw, Poland; agata_antoniewska@sggw.edu.pl (A.A.-K.); jaroslawa_rutkowska@sggw.edu.pl (J.R.); 5 Faculty of Veterinary, Universidad de Zaragoza, C/Miguel Servet, 177, 50013 Zaragoza, Spain

**Keywords:** *Tenebrio molitor* oil, volatile compounds, sensory aroma attributes, phenolic compounds, antioxidant properties, fatty acids, carotenoids, tocopherols

## Abstract

During defatted *Tenebrio molitor* (TM) larvae powder production, oil is obtained as a by-product, mainly intended for feed enrichment or as a biofuel component. In 2021, EFSA authorized TM as the first insect to be a novel food. Thus, the study aimed to assess the composition, including fatty acids (FAs), tocopherols, carotenoids, phenolics, volatiles, antioxidant capacity, sensory aroma attributes, physical properties, and oxidative and hydrolytic stability of TM oil. The FAs profile was dominated by oleic—C18:1*9c* (36.8%) and linoleic—C18:2*9c12c* (32,4%) acids, resulting in a PUFA/SFA ratio similar to vegetable oils. Thus, TM oil was characterized by a beneficial Health Promoting Index (HPI) (2.42), which was 10-fold higher than the HPI of common animal fats. TM oil contained bioactive compounds such as carotenoids (13.65 mg/kg), tocopherols (105.8 mg/kg), and phenolic compounds (74 mg GAE/kg). A noticeable amount of apigenin was also noted among nine detected phenolic compounds. The substantial presence of lipophilic and phenolic compounds contributed to antioxidative potential. Sensory estimation revealed the dominance of fried and nutty aromas, probably because of the abundance of Strecker aldehydes and pyrazines in their volatile profile. The results indicated that the technological process needs modification to limit the formation of lipid oxidation volatile compounds such as aldehydes and eliminate some differences between batches. This preliminary study on the composition and properties of TM oil encourages its use as an ingredient for food, pharmaceutical, and cosmetics purposes.

## 1. Introduction

The use of edible insects has frequently been linked to their use as feed for animal consumption. However, in recent years, due to the increase in world population and the growing demand for proteins of animal origin in developing countries, its possible use as food for human consumption has acquired great relevance, representing one of the possible responses with lower environmental impact [1]. The Food and Agriculture Organization (FAO) has remarked that insects are a very nutritious and healthy food source with high protein content and good nutritional value [2]. Additionally, its production entails a smaller ecological footprint and is more sustainable than conventional livestock farming since it produces less greenhouse gases, requires less land and water use, and its feed can be based on byproducts [3,4].

Although demand for insect products is expected to increase greatly in the next decades, its consumption as a whole dried insect in Western countries is not widely accepted among consumers, mainly for cultural reasons. Therefore, the introduction into the European market should focus on products derived from insects as ingredients, in which the anatomy of the animal is not perceived, such as flour or oils.

It is estimated that there are more than 2000 species of edible insects that have traditionally been consumed throughout the world. Among them are the larvae of *Tenebrio molitor*, also called yellow mealworm. This insect has aroused special interest due to its rapid production and nutritional value since it has a very high content of proteins (52%) with high biological value and unsaturated lipids, compared with other insects, as well as other interesting components such as antioxidant compounds, chitosan, or bioactive peptides [5,6,7].

Yellow mealworm was the first insect species to receive a positive opinion from the European Food Safety Authority (EFSA) as a novel food in 2021 [8]. Currently, frozen and dried yellow mealworms (whole or powder) commercialization is allowed in the European market [9]. Interest in *Tenebrio molitor* has been mainly focusing on its protein fraction value, such as defatted mealworm powder, and its potential uses for feeding animals, but more recently for human consumption as a bakery ingredient or protein bars [10]. Defatted mealworm powder is also a potential functional ingredient in food production due to its high antioxidant capacity [11].

During the production of defatted mealworm powder, *Tenebrio molitor* oil is obtained as a by-product. Different methods could extract this oil. The traditional and most common method is the mechanical pressing of dried larvae. However, this method requires further refining steps to avoid solid residues of undesirable particles present in the oil. Alternative approaches, such as organic solvents or supercritical carbon dioxide extraction, have been employed [12] to improve efficiency.

The previous study indicated insect oil as a source of unsaturated fatty acids, including essential belonging to ɷ-6 and ɷ-3 families and certain bioactive compounds with antioxidant properties, such as tocopherols and polyphenols [12]. Thus, insect oils can be considered an alternative source of valuable lipids in human nutrition [6,12]. The potential benefit of insect oil use in human consumption has not been widely studied, and its production is mainly intended for feed enrichment or as a biofuel component despite its low toxicity and interesting nutritional profile [13]. The results of a study by Lee et al. [7] suggest a suppression of hyperlipidemia and hepatic steatosis in mice in which part of the fat in their diet had been partially replaced with *Tenebrio molitor* oil [7]. Other studies suggested the antimicrobial activity of the oil, retarding the growth of both Gram-positive and Gram-negative pathogenic bacteria and improving the intestinal barrier and maintenance of intestinal health in rabbits [14]. These results point to possible health benefits if this type of oil is incorporated into the human diet. However, studies devoted to assessing the aroma attributes of *Tenebrio molitor* oil are scarce. A previous study by Lee et al. [15] presented the volatile composition of the oil of various insect larvae, indicating 2-methylbutanol and pyrazine compounds as characteristic of their aroma. Regarding the proposal of novel food, the knowledge of sensory attributes is necessary to recognize sensory profiles and predict consumer acceptability. A study by Perez-Santaescolastica et al. [16] is a valuable, comprehensive review that summarizes studies on the sensory properties of edible insects and edible insect-based products. However, there is no research on sensory estimation of aroma attributes of *Tenebrio molitor* oil. Thus, the study aimed to assess the profile of bioactive compounds (including fatty acids (FAs), tocopherols, carotenoids, and phenolics), antioxidant capacity, oxidative and hydrolytic stability, and physical properties to assess the potential of *Tenebrio molitor* oil as a potentially valuable food ingredient. Complementarily, volatile profiles and sensory estimation were studied to describe aroma attributes and recognize and understand compounds involved in the aroma of *Tenebrio molitor* oil.

## 2. Materials and Methods

### 2.1. Preparation of Yellow Mealworm Oil

**Figure 1:**
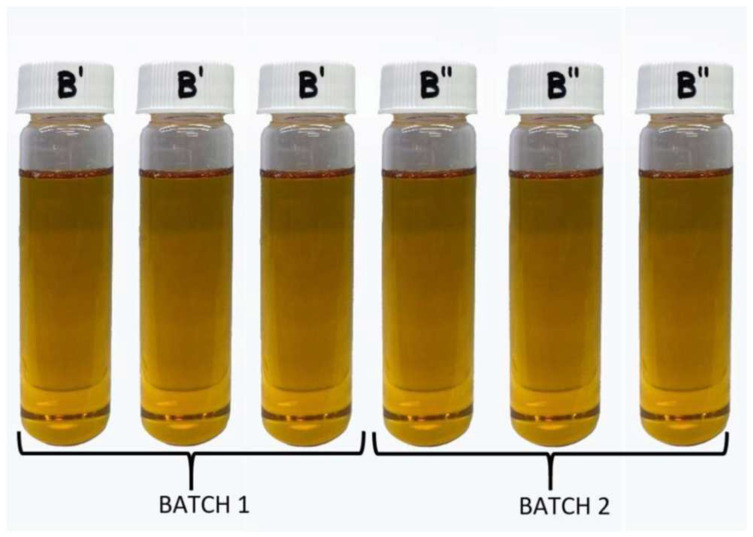
Analyzed samples of *Tenebrio molitor* (TM) oil derived from two production batches. B’: samples replicates from Batch no.1, B”: samples replicates from Batch no.2.

The breeder reared the larvae to Bugcle Bioindustrias, SL. (Huesca, Spain). *Tenebrio molitor* (TM) larvae were kept for a period of 16–18 weeks in a rearing room in conditions proposed in the previous study [17]. They were fed ad libitum with wheat bran and carrot. Mealworms were fasted for 3 days to empty their guts and then separated from the frass and washed with water. Next, larvae were blanched in boiling water (for 3 min) and then rack-oven dehydrated (at 75 °C for 6 h). An automatic oil screw press (ERNP^TM^) was applied to crush dehydrated larvae to obtain two different fractions: the oil and the defatted solid fraction (flour). The oil fraction was then collected in a stainless steel tank and passed through a plate filter to eliminate impurities. Finally, the oil was bottled as the final product. The experiment included two industrial productions. Three samples from each production batch were taken for analysis. Experimental samples are presented in Figure 1.

### 2.2. Determination of Oxidative and Hydrolytic Changes in Oil Samples

Total free fatty acids (FFAs) of TM oil samples were determined by titration, according to the standard method [18], and expressed as a percentage of free fatty acids (% FFA).

TM oil samples were analyzed for lipid oxidation by determining primary products using peroxide value (PV), expressed as milliequivalent active oxygen per kg of oil (mEq O active/kg oil). PV was determined according to the standard volumetric titration method [19].

The contents of conjugated dienes (CD) and conjugated trienes were determined at 232 nm and 270 nm wavelength using UV-Vis spectrophotometer (insert spectrophotometer data) according to the method of ISO 3656:2011 [20]. The 1% (*w*/*v*) oil solution absorbances in cyclohexane (Panreac Química S.L.U., Barcelona, Spain) were measured [20].

Oxidative stability of TM oil samples was expressed as the oxidation induction time (hours), measured with a Rancimat^TM^ 743 instrument (Metrohm, Herisau, Switzerland), using 3 g of oil warmed to 120 °C with 20 L h^−1^ airflow. Induction time is the time required to reach the breaking point of the curve.

### 2.3. Analysis of Fatty Acid Profile

The fatty acids (FAs) profile of TM oil samples was determined as fatty acid methyl esters (FAMEs) using gas chromatography (GC) following the method described by Mallor et al. (2023) [21]. Transmethylation of TM oil samples was conducted using sodium methoxide in methanol and acetyl chloride in methanol (1:10, *v*:*v*) (Panreac Química S.L.U., Barcelona, Spain). Separation of FAMEs was conducted using a Bruker 436 Scion instrument (Bruker, Billerica, MA, USA) equipped with a flame ionization detector, with a CP-8400 autosampler and an SP-2560 capillary column (100 m × 0.25 mm ID × 0.20 μm) (Supelco, St. Louis, MO, USA). The detailed conditions of chromatographic separation are presented in the study of Baila et al. [22]. Peaks were identified by comparison with the relative chromatographic retention times of the standard FAME mixtures GLC-401, GLC-532, GLC-538, GLC-643, and GLC-642 (Nu-Chek Prep, Elysian, MN, USA). Quantification of FAMEs was achieved in relation to the internal standard nonadecanoic acid (C19:0) (N-19-M Nu-Chek Prep, Inc., Elysian, MN, USA), which was added before the transmethylation of oil samples. The contents of individual FAs were expressed as g per 100 g of total FAs.

#### 2.3.1. Oxidizability Value (COX)

Oxidizability value (COX), to determine the effect of the fatty acid composition, was also calculated using the method described by Fatemi and Hammond [23], as shown below:

COX = (1 × (C16:1 + C17:1 + C18:1 + C20:1) + 10.3 × (C18:2) + 21.6 × (18:3 + C20:3))/100, (1)

where C represents the percentage of each fatty acid.

#### 2.3.2. Nutritional Quality Index Determination

To evaluate the influence of the fatty acid composition of the oils on the risk of developing cardiovascular disease, three different indexes were calculated: thrombogenicity index (TI); atherogenicity index (AI); and the ratio of hypocholesterolemic to hypercholesterolemic FA (HH). The calculations were performed according to the formulas described by Ulbricht [24] and Santos-Silva et al. [25]. The health-promoting index (HPI) was proposed by Chen et al. in 2004 [26]:

(2) $$\mathrm{TI}=\frac{C14\text{:}0+C16\text{:}0+C18\text{:}0}{\left(0.5\;\times \text{Ʃ}\mathrm{MUFA}\right)+\left(0.5\times \text{Ʃ}ɷ6\right)+\left(3\times \text{Ʃ}ɷ3\right)+(\frac{ɷ3}{ɷ6})}$$

(3) $$\mathrm{AI}=\frac{C12\text{:}0+\left(4\;\times C14\text{:}0\right)+C16}{\text{Ʃ}\mathrm{MUFA}+\text{Ʃ}ɷ3+\text{Ʃ}ɷ6}$$

(4) $$\mathrm{HH}=\frac{C18\text{:}1+C18\text{:}2+C18\text{:}3+C20\text{:}4+C20\text{:}5+C22\text{:}5+C22\text{:}6}{C14\text{:}0+C16\text{:}0}$$

(5) HPI=ΣUFA/(C12:0+(4 × C14:0)+C16:0)

### 2.4. Assessment of Phenolic Compounds

#### 2.4.1. Total Phenolic Compounds (TPCs)

The TPC was assayed by a spectrophotometric method using the Folin–Ciocalteu reagent at 765 nm (Jasco V-530, Easton, MD, USA/Madrid, Spain) according to the method proposed by Capannesi et al. (2000) [27]. The results were expressed as gallic acid equivalents (GAE; mg GAE/kg of sample).

#### 2.4.2. Phenolic Compounds Profile

The qualitative and quantitative determination of phenolic compounds extracts was conducted using HPLC-HRMS analysis. The equipment used was the chromatography fraction of Thermo Scientific Ultimate 3000 coupled to Orbitrap Fusion Tribrid Series HRMS analyzer (Thermo FisherScientific, San Jose, CA, USA). Chromatographic separations were executed using the column Luna C18 (2.1 × 150 mm, 3 mm) (Phenomenex, Bologna, Italy) with a constant temperature of 40 °C and a 0.2 mL/min flow rate. A binary mobile phase was A (water–formic acid; 0.1% *v*/*v*) and B (acetonitrile–formic acid) (ITW Reagents, S.R.L., Milán, Italy). The elution procedure was performed as follows: time/[A:B], 0 min/[100:0], 4 min/[95:5], 35 min/[0:100], 37 min/[0:100]. The sample solution injection volume was set to 20 μL. For mass spectrometry, the ion source was an H-ESI working in the positive ion mode, in the full-scan mode range of 100–1000 *m*/*z*, with a resolution of 30,000 in FTMS. Xcalibur 4.0 software (Thermo Scientific, Bremen, Germany) was used for acquisition, data evaluation, elaboration, and calculation. Quantification was based on internal calibration curves built using the following standard compounds: malvidin, chlorogenic acid, apigenin, epicatechin, naringenin, quercetin, gallic acid, ferulic acid, and coumaric acid.

### 2.5. Total Carotenoids Determination

Carotenoid content was measured spectrophotometrically using the method proposed by Motilva et al. [28]. Oil samples were dissolved in cyclohexane (O:C) (Panreac Química S.L.U., Barcelona, Spain) until a final volume of 3 mL and analyzed spectrophotometry at 472 nm with a Jasco UV-Vis Spectrometer (Easton, MD, USA/Madrid, Spain). The concentration of carotenoids was calculated by applying the following formula:

C = [(E × Vf)/(E_1%_ × W)] × 10,000 (6)

where C was the final pigment concentration (mg of carotenes/kg of oil); E was the absorbance measured at the corresponding λ; Vf was the final volume of pigment extract (mL); E_1%_ was the specific absorbance of a 1% solution measured in a 1 cm cuvette (E_1%_ = 2000); and W was oil sample weight (g).

### 2.6. Tocopherols and Cholesterol Determination

Tocopherols and cholesterol in *Tenebrio molitor* larvae oils were extracted according to Rufino-Moya et al. (2020) [29] with some modifications. About 100 mL of the samples were saponified overnight with 3 mL of a solution of ascorbic acid and 10% potassium hydroxide (KOH) (Panreac Química S.L.U., Barcelona, Spain) in ethanol–distilled water (50:50, *v*:*v*) under a nitrogen atmosphere; then, the lipophilic compounds were extracted twice with 5 mL of n-hexane–ethyl acetate (9:1, *v*:*v*, 5 μg·mL^−1^ of BHT) solution (Panreac Química S.L.U., Barcelona, Spain). After this, the supernatants were evaporated in a vacuum evaporator. The dry residues were dissolved in 1 mL of acetonitrile–dichloromethane–methanol (75–10–15, *v*:*v*:*v*) (Panreac Química S.L.U., Barcelona, Spain), filtered through a 0.22 mm polytetrafluoroethylene (PTFE) filter, and transferred into a 2 mL amber glass vial for automatic sampling using 5 mL for ultra-performance liquid chromatography (UPLC).

The chromatographic and quantification procedure was used by Chauveau-Duriot et al. (2010) [30] into an ACQUITY UPLC H-Class liquid chromatograph (Waters, Milford, MA, USA), coupled with a fluorescence detector (2475 Multi l Fluorescence Detector; Waters) and controlled by Empower 3 software (Waters). Separation was carried out on an Acquity UPLC HSS T3 column (150 mm × 2.1 mm × 1.8 mm × 2.1 mm; Waters). Detection of tocopherol homologs was undertaken by fluorescence under an excitation wavelength of 295 nm and emission of 330 nm. Identification of chromatographic peaks was based on retention times by comparison with known standards (99% purity α-tocopherol, 97% purity γ and δ-tocopherol) purchased from Sigma-Aldrich (St. Louis, MO, USA). Concentrations of α, γ, and δ-tocopherol standard solutions were calculated before use by absorbance of each solution using molar absorption coefficients, as previously reported [20]. An external calibration curve was prepared for each tocopherol standard to calculate the amount of tocopherol in the oil sample.

### 2.7. Antioxidant Capacity

Antioxidant capacity was determined by measuring DPPH radical scavenging activity and ABTS radical scavenging activity. The DPPH radical scavenging activity determination of the whole oil was performed according to the method proposed by Tuberoso et al. (2007) [31] with some modifications. The oil sample (100 µL) was incubated with a DPPH ethyl acetate solution (Sigma-Aldrich (St. Louis, MO, USA) (3 mL, 0.05 mM) for 60 min in darkness at room temperature. The decrease in absorbance of the resulting solution was measured at 517 nm, with a Jasco UV-Vis Spectrophotometer (Easton, MD, USA/Madrid, Spain).

The ABTS radical scavenging activity was measured according to modified methods [32,33] to evaluate the antioxidant activity of the polar part of the oil. Briefly, 2.5 g of oil sample was taken in a centrifuge tube, 2.5 mL of methanol (Panreac Química S.L.U., Barcelona, Spain) was added, and vigorously mixed with vortex and centrifuged at 3600× *g* for 5 min (Orto Alresa Digicen 20-R; Madrid, Spain). The supernatant was collected and the polar extract was obtained. The extraction with methanol (2.5 mL) (Panreac Química S.L.U., Barcelona, Spain) was repeated three times.

ABTS radical cation (ABTS^+•^) Sigma-Aldrich (St. Louis, MO, USA) was previously prepared by reacting 5 mL of stock solution (7 mM) with 5 mL of 2.45 mM potassium persulfate solution and stored in darkness for 16 h. Then, ABTS^+•^ solution was diluted with methanol (A734 = 0.700 ± 0.02). An aliquot of 100 µL of oil polar extract was mixed with 3 mL of ABTS^+•^ solution. The mixture was vortexed for 10 s, and the absorbance (λ = 734) measurement was performed after 15 min in darkness at room temperature with a Jasco UV-Vis Spectrophotometer (Easton, MD, USA/Madrid, Spain).

In both methods, a Trolox standard curve in the range 0.1–0.4 mM was prepared, and the results were expressed as Trolox equivalent antioxidant capacity per kg (mM TEAC/kg) and as % Inhibition using the following equation:

(7) $$\%\mathrm{Inhibition}\;=\;\frac{{(A}_{b}-\;A_{t})}{A_{b}}\;\times \;100$$

where A_b_ was the blank sample’s absorbance and A_t_ the analyzed sample’s absorbance.

### 2.8. Analysis of Volatile Compound Profile

The headspace solid-phase microextraction (HS-SPME) technique was applied before gas chromatography–mass spectrometry (GC-MS) to determine the volatile compounds of *Tenebrio molitor* larvae oil samples. For HS-SPME extraction of volatiles from 6 g of oil sample, a triple-coated fiber DVB/CAR/PDMS (divinylbenzene/carboxy/polydimethylsiloxane; 10 mm length, 50/30 mm thickness, Supelco, Bellefonte, PA, USA) was used. The fiber was exposed to the sample’s headspace for 40 min at 45 °C. Chromatographic separation was performed on a GC-MS instrument (6890N GC, 5975 MS Agilent Technologies, Santa Clara, CA, USA) equipped with an HP-5MS column (30 m × 0.25 mm × 0.25 μm; 5%-diphenyl-95%-polydimethylsiloxan; Agilent, Santa Clara, CA, USA). The detailed conditions of the oven temperature program were described previously [34]; the carrier gas was helium at 0.9 mL/min, splitless injection. The MS was operated at 70 eV, source temperature at 230 °C, and mass range 33–350 *m*/*z*. The identification of the volatile compounds was based on comparing the Kovats’ retention indexes and mass spectrum with those of NIST.08 Mass Spectral Search Program and Wiley 8th Ed. libraries. The determination of the linear retention indices (LRIs) was performed, using a mixture of n-alkanes (C6–C20; Sigma-Aldrich, Poznań, Poland) dissolved in n-hexane, according to Van del Dool and Kratz (1963) [35]. The quantities of volatile compounds were presented as a relative percentage of the total peak area [34]. The analyses were carried out in triplicates.

### 2.9. Analysis of Physical Properties

The surface tension of the oils was determined by the Du Noüy ring method using a K6 tensiometer (Kruss, Hamburg, DE) at 25 °C.

The oils’ viscosities were determined using a Brookfield CAP-2000+ rotational cone–plate viscometer equipped with a Peltier thermoelectric controller and CAP-CALC/CAP266Y (v. 3) software (AMETEK Brookfield, Middleboro, MA, USA). Tests were performed at 25 °C and with a shear rate of 100 s^−1^.

### 2.10. Sensory Evaluation of Aroma Attributes

The sensory assessment of aroma attributes of *Tenebrio molitor* larvae oil samples was conducted by 13 experienced panelists aged 25–60 years. Among these, 44% of the panelists were male and 56% female. The panelists evaluated the intensity for each aroma attribute using a five-point structured scale: 0—very faint; 2—slightly; 4—moderate; 6—intense; 8—very intense. Each panelist smelled the two different samples coded with random three-digit numbers. Panelists were requested to evaluate the intensities of the aroma attributes: fried, nutty, woody, earthy, roasted and rancid. The rating was conducted three times. The assessments were carried out in the sensory laboratory room, fulfilling the requirements of the ISO standard. Before participating in the evaluation, all assessors read the information sheet and signed their informed consent form stating they had no allergies to shellfish or house dust mites. Participants knew that the study aimed to determine the aroma attributes of oil obtained from *Tenebrio molitor* larvae.

### 2.11. Statistical Analysis

Analysis was conducted in triplicate in both oil batches. Results are presented as mean ± SD. The measurements obtained for different parameters were analyzed using one-way ANOVA followed by Tukey’s post hoc test, and values of *p* < 0.05 were considered significant. Statistical analyses were performed with GraphPad Prism 5.0 software (GraphPad Software, Inc., San Diego, CA, USA).

## 3. Results and Discussion

### 3.1. Oxidative and Hydrolytic Stability of Tenebrio molitor Oil

The TM oil samples derived from two batches of production did not differ significantly (*p* < 0.05) in the oxidative and hydrolytic quality, except for the peroxide value—PV, which was slightly higher in a sample of Batch no. 1 (Table 1). PV is the most common chemical indicator for measuring the oxidative deterioration of oils. Although hydroperoxides decompose to a mixture of volatile and non-volatile products and react further to endoperoxides and other products, PV measurement is a useful method of monitoring the oxidative deterioration of oils. Samples derived from both production exceeded the limit of <10 mEq/kg oil considered appropriate for fresh oils but still remained far from the 20 mEq/kg from which rancidity begins to be perceived in many oils [36]. Compared with PV values, the contents of conjugated diene hydroperoxides related to oxidative changes of linoleic acid C18:2*9c12c* were relatively low (Table 1). Also, low amounts of conjugated trienes were assayed probably because of low amounts of linolenic acid—C18:3*9c12c15c* were assayed in the FAs profile of TM oil samples (Table 2). However, our results show that raw oil values are higher than expected. However, this result could be easily related to the high polyunsaturated profile of the TM oil. The induction time observed, which refers to the period of time before the chain reaction of oil oxidation begins to accelerate, is considerably short, less than 3 h. These oxidation stability results are lower than those previously shown in other *Tenebrio molitor* oil studies, which varied between 10:56 and 64:57 h [37,38] and closer to those obtained in seed oils, also very polyunsaturated [39]. The low oxidative stability indicates the need to carefully treat the product before consumption to maintain its nutritional and organoleptic properties.

The formation of FFA is an effect of the hydrolytic decomposition of triacylglycerols in oils and fats. FFA levels depend on time, temperature, and moisture content because the oils and fats are exposed to various environments such as storage, processing, heating, or frying. In the case of insect oil, FFA content results from the enzymatic action causing initial hydrolysis or lipolysis by the lipases present in the living tissue of the mealworm. The dynamic equilibrium between the proportion of triacylglycerols and FFA might be affected by the physiological state of the mealworm [40]. Results obtained in this study were lower than those previously reported by Son et al. (2020) (2.6 mg KOH/g) in TM oil [38].

### 3.2. Fatty Acids Composition and Nutritional Quality Index of Tenebrio molitor Larvae Oil

The application of high-resolution GC enabled the detection of 22 fatty acids (FAs) in TM oil samples (Table 2). Despite being an animal-origin oil, the unsaturated fraction of TM oil samples was predominant, with almost 75% of total FAs. The major FAs were oleic (C18:1*9c*), linoleic (C18:2 *9c12c*), and palmitic (C16:0) acids, respectively. These results are in accordance with previous studies made on TM oil [38,41].

PUFA/SFA is an index used to assess the impact of diet on cardiovascular health. PUFAs in the diet are associated with depressed low-density lipoprotein cholesterol (LDL-C) and lower serum cholesterol levels. In contrast, some SFAs (palmitic and myristic) contribute to high serum cholesterol levels. Thus, a higher ratio of this type is beneficial for preventing cardiovascular disease. The results showed a higher PUFA/SFA proportion in TM oil than those found in lipids extracted from meat (0.11–0.95) and dairy products (0.06–0.17), and more similar to fish (0.51–1.79), shellfish (0.20–2.10), and certain brown and red seaweeds, although higher than green ones (0.44–0.88) [42]. The PUFA group was represented mainly by two compounds: linoleic C18:2*9c12c* (32.44%) and α-linolenic C18:3*9c12c15c* (1.46%). Substantial presence in TM oil of α-linolenic is beneficial regarding human health and well-being and was in accordance with previous results (1.22–1.74%) [36,41]. The results of the ɷ-6/ɷ-3 ratio were lower than other studies, which indicates that in *Tenebrio molitor* larvae, this ratio ranges between 1:31.5 and 1:40 depending on diet formulation [43]. The effect of dietary fatty acids profile on cardiometabolic risk factors and the risk of developing some pathologies have been widely demonstrated [44]. Ingestion of an appropriate balance of ɷ-6/ɷ-3 ratio suggested as 4:1 has been widely associated with a reduction risk of many pathologies like cardiovascular disease, cancer, inflammatory bowel disease, rheumatoid arthritis, and bone disease, among others [45]. Although with a typical flour-based diet, the ɷ-6/ɷ-3 ratio is higher than recommended, it has been observed that including ingredients rich in ɷ-3, like linseed, in larvae feed could easily improve this ratio [46,47]. Moreover, α-linolenic acid is a precursor of eicosapentaenoic acid C20:5 and docosahexaenoic acid (C22:6); thus, the TM oil can be considered a sustainable source of valuable FAs [48].

Regarding reducing the risk of coronary heart disease, it should be mentioned that a lower amount of cholesterol is assayed in TM oil (146 mg/100 g) than in many common animal-derived sources (eggs, pork, and butter) (Table 2) [49,50]. A previous study also revealed that specific feeding regimens may provide an even lower cholesterol level than assayed in our study [7].

Indexes were calculated to evaluate TM oil’s nutritional quality FA profile (Table 2). The two first indexes, thrombogenicity (TI) and atherogenicity (AI), indicate whether TM oil has the potential to stimulate platelet aggregation. The lower the TI and AI values, the more anti-atherogenic fatty acids are present, which means that there is greater potential for preventing the development of coronary heart disease. According to previously reported values, the obtained results in TI and AI, 0.61 and 0.41, respectively, were lower compared with values of indexes of some other animal origin products, such as milk products, lamb, beef or pork meat, and chicken, which is considered as white meat and healthier, whose range varied from 0.79 to 2.07 for TI, and 0.50 to 2.03 for AI [24,41]. Results were even better than some vegetable margarines, especially the TI, which was 2-fold lower in TM oil. Similar values of AI were found in avocado oil [51]. The specific effects of fatty acids on cholesterol metabolism are evaluated by the hypocholesterolemic–hypercholesterolemic (HH) index. In this case, high HH values are desired from a nutritional standpoint. Results showed a higher HH index in TM oil than in vegetable oils like avocado fruit (2.06) [48].

Health-promoting index (HPI) was proposed to assess the nutritional value of dietary fat, which focuses on the effect of FA composition on cardiovascular disease. Higher HPI values are considered to be beneficial to human health. Results indicate a 10-fold higher index in comparison with HPIs of other animal fats, like dairy products [42], but also higher than previously published in the literature about TM oil (1.38) [52].

Oxidizability value (COX) is a parameter that evaluates the oil’s tendency to undergo autoxidation. The rapidity of oxidation depends on the degree of unsaturation, the presence of antioxidants, and the prior storage conditions. Compared with literature data, TM oil showed a better autoxidation tendency than vegetable edible oils rich in unsaturated FAs, such as sunflower oil (7.25), peanut oil (4.63), or certain seed oils, but lower than previously reported for extra virgin olive oil (2.38) [39,53].

### 3.3. Bioactive Components and Antioxidative Potential of Tenebrio molitor Larvae Oil

The content of different compounds that contribute to antioxidative oil properties is shown in Table 3. The total tocopherol content obtained was 106 mg/kg, being α-tocopherol the most abundant, accounting for 52.74% of the total tocopherol (55.8 mg/kg), followed by γ-tocopherol, which represented 44.23%. Significant (*p* < 0.05) differences were observed between batches. Tocopherols (vitamin E) are a natural antioxidant that prevents free radical and hydroperoxy radical oxidation of lipids, one of the most important nutrients in fat-based products. The results of our study confirmed that TM oil can be regarded as a valuable source of tocopherols. Previous results indicated a higher total tocopherol content (144–195 mg/kg), and γ-tocopherol as the main one, while α-tocopherol content was similar to our results [37,38]. Different aspects, such as the larvae feeding the oil extraction method, could explain these disparate results. Ingredients are determinant for the contribution to tocopherol concentrations. It has been previously observed in other animals that feed concentration of tocopherols is correlated with the final meat tissue concentration [54,55]. Furthermore, it has been stated that the larvae processing method and oil extraction procedure determine the oil’s final nutritional composition and tocopherol content [12]. In the Jeon et al. (2016) study, larvae were freeze-dried, grinding, and then extracted with solvents (n-hexane) [37], while Son et al. (2020) blanched mealworms, then dehydrated with hot air, ground, and finally extracted the oil also using solvents (n-hexane) [38]. Our study samples were also submitted for blanching, followed by dehydration with hot air, but the oil was obtained directly by cold pressing of the dehydrated larvae.

Particularly noteworthy is the carotenoid content, 13.65 mg/kg oil. It was attributed to a feeding regimen of larvae rich in carotenoids because they contained carrots. Carotenoids, especially β-carotene, have antioxidant properties, are a precursor of Vitamin A, and impart the orange color in some food products. Due to its chemical structure, it quenches singlet oxygen with a multiple higher efficiency, 2.5–3 fold, than α-tocopherol, showing relevant potential antioxidant biological properties [56]. It should be noticed that carotenoids were not previously assayed.

Analyzed in our study, TM oil samples were also distinguished by substantial content of total phenolic compounds (TPCs)—74.09 mg GAE/kg, considerably higher than previously reported (18 mg GAE/kg) for TM oil [38]. It should be remarked that this content differs significantly from that shown by plant oils rich in polyphenols, like olive or rice brands, which mostly show higher contents, especially those virgin or obtained by cold-press methods [57]. However, the content of total phenolic content in plant oil, which shows large differences between types, depends on many factors, including the condition of the raw material and its variety, degree of its maturity, cultivation (i.e., weather conditions, agrotechnical operations), as well as methods of extraction and purification of oil [57]. These facts could explain the similar or even 4-fold higher total phenolic content observed in TM oil compared with other cold-press plant oils extracted in similar conditions, such as camellia seed oil, pistachio nuts oil, or pumpkin seed oil, among others [58,59,60].

It should also be noted that according to previous literature, whole-dried TM larvae show higher total phenol contents, ranging between 350 and 920 mg/100 g [61,62,63]. In comparison, other authors indicated that the contents of defatted TM flour should be around 200 mg/100 g [11]. Phenols include one or more hydroxyl groups, the polar part, attached directly to an aromatic ring, the nonpolar part. They are often found in plants as esters or glycosides, rather than as free molecules. This stereochemistry distinguishes phenols according to their polarity variance [64] and can determine their affinity for the different fractions of *Tenebrio molitor*-derived products, defatted flour, or oil. Chromatographic (HPLC-HRMS) analysis enabled the detection of apigenin in TM oil samples (Table 3). Apigenin (4′,5,7-trihydroxyflavone), a hydrophobic flavonoid that presents low solubility in both polar and nonpolar solvents, also occurs naturally in a wide variety of edible plants and fruits, like onion, seasoning herbs, pistachio nuts, beers, or olive oil, among others [65,66]. Literature has related apigenin to many health-beneficial properties, i.e., it is a potent antioxidant, antiviral, or therapeutic aid in cancer treatments [67,68,69].

Apart from detecting apigenin, HPLC-HRMS chromatographic analysis revealed the trace presence of other dietary bioactive compounds like fraxin, geranylgeraniol, or β-cymaro pyranose. Fraxin, a hydroxycoumarin derivative, exhibits health benefits like potent hepatoprotective effects in vitro and in vivo [70] and action as an anti-inflammatory agent [71,72]. Geranylgeraniol, an isoprenoid also found in fruits, vegetables, grains, and edible plant oils with health benefits, has improved glucose homeostasis, lipid metabolism, osteogenesis, and bone remodeling in animals [73,74]. However, these eight phenolic compounds were detected under the limit of quantification (LOQ)—0.10 mg/L (Table S1). Using an appropriate mixture of solvents will probably allow us to obtain higher amounts of these valuable phenolic compounds in TM oil.

Significant (*p* < 0.05) differences in the contents of bioactive compounds between oil samples derived from two batches were stated (Table 3). It may have resulted from differences in feed intake by individuals of larvae during their rearing. It also may be derived from differences in the growth rate. Thus, the concentration of carotenoids from carrots from the feed may substantially differ in the larvae body. The importance of the feeding regimen on the larvae composition was comprehensively revised elsewhere [75].

ABTS and DPPH radical cation scavenging assays were carried out to determine the antioxidant potential of the yellow mealworm oil. Both methods indicated % of inhibition between 35% and 41% (Table 3). Previous studies have shown lower values (10–15%) in hot air-dried yellow mealworm larvae [62]. Ugur et al. (2020) reported values of 0.17 mg Trolox/g in TM oil extract at an atmosphere pressure of 30 °C, also lower than the results of this study [76]. Its antioxidant properties are probably attributed to different molecules present in the oil. It is estimated that the main polyphenol found, apigenin, possesses a potent antioxidant action, with an IC50 radical cation scavenging of 0.575 and 0.344 mg/mL for DPPH and ABTS assay, respectively [74], which partially explain the antioxidant capacity observed.

Phenolics are not the only antioxidant compound assayed in TM oil. ABTS and DPPH radical scavenging activity methods are reactive towards many different types of molecules, not only polyphenols. As was mentioned earlier, TM oil samples contained substantial amounts of non-polar compounds such as carotenoids and tocopherols that contribute to the antioxidant potential. TM oil may partially replace vegetable oils because it is a source of many bioactive compounds and has high antioxidative potential.

### 3.4. Volatile Compounds

A total of 68 different compounds were identified in the headspace of TM oil samples, including aromatic hydrocarbons (18), aldehydes (13), hydrocarbons (9), pyrazines (7), ketones (6), terpenes (5), carboxylic acids (3), and furans (1) (Table 4).

Regarding total relative amounts, aldehydes (54.36%) were the most abundant volatiles in analyzed samples of TM oil. Most of the identified aldehydes are associated with the characteristic classes of secondary oxidation products of PUFAs, resulting mainly from autoxidation of linoleic—C18:2 *9c12c*, α-linolenic—C18:3 *9c12c15c* and oleic—C18:1 *9c* acids. Hexanal was, by far, the main volatile compound detected in the analyzed TM oil. This aldehyde and 2-hexenal may be a heating-induced oxidation product of FAs (C18:2 *9c12c*, C18:3 *9c12c15c*). Probably, the dominant share of linoleic acid (32.44%) has the most influence on the formation of hexanal in analyzed samples of TM oils (Table 4).

Formation of other aliphatic aldehydes, such as pentanal, heptanal, octanal, nonanal, 2-butyl-2-octenal, and 2-hexenal, was caused by autoxidation of unsaturated 18 FAs (Table 4) [77]. These aldehydes were also previously reported in TM of larvae and obtained larvae oil [77]. It should be noted that volatile secondary and tertiary degradation products formed during lipid autoxidation are responsible for certain (off) flavor attributes in food. In the case of hexanal, its odor threshold is 4.5 ppb, and its sensory attribute is fatty, oily, grassy, tallow, and rancid but also apple and green [78,79,80].

Hydrocarbons were numerously represented in TM oil samples by 8 and 18 compounds belonging to aliphatic and aromatic hydrocarbons, respectively (Table 4). Their presence in TM oil samples probably resulted from lipid oxidation. Many were formed as tertiary lipid oxidation products from aldehydes due to high-temperature exposure during the technological process (75 °C). As stated in a previous study, some aromatic hydrocarbons, e.g., toluene and xylene, may derive from the decomposition of carotenoids, for which a substantial amount was assayed in our study (Table 4) [81]. Lee et al. (2022) also detected some of the hydrocarbons of our samples: undecane, ethylbenzene, toluene, o-xylene, and p-xylene [15]. However, hydrocarbons do not significantly contribute to TM oil’s aroma because many are odorless or possess high odor thresholds [16,34]. Among the three detected alcohols, the presence of 1-octen-3-ol is worth mentioning. This compound was described to significantly affect the aroma by contributing to a characteristic mushroom aroma [16].

A substantial abundance of methyl ketones also characterized the volatile profile of TM oil samples; in total, six compounds were detected (Table 4). These compounds may be formed from unsaturated FAs and their unsaturated secondary degradation products resulting from lipid oxidation. They can be considered tertiary products of lipid oxidation [32,81]. Ketones are odor-active; three of them: 2-butanone, 2-heptanone, and 2-decanone may contribute to the fruity, ethereal, floral, and fatty aromas of edible insects [81]. Carboxylic acids represent a higher relative abundance than ketones (6.91%); however, the aromatic contribution of this group is mainly undesirable, as their odors are described as acid, sharp, pungent, fecal, and sweaty (acetic acid, 3-methyl butanoic acid, and hexanoic acid) [81]. However, significant differences in the relative abundance of carboxylic acids between batches should be noted, which may result from differences in the physiological state of some larvae individuals as compared with the majority of larvae used in oil production [75].

Before oil extraction, larvae are subjected to a drying process. During this process, the Maillard reaction occurred. Some of the volatile compounds, formed during the degradation of branched-chain amino acids and associated with this browning process, like 2-methylpropanal, 3-methylbutanal, 2-methylbutanal, but also methylpyrazines, were developed and partially transferred to the oil [80]. Keil et al. (2022) reported the presence of these compounds in significantly higher content in mealworm larvae dried in a rack oven at 60 °C [77]. Pyrazines detected in TM oil’s volatile profile are compounds formed in the Maillard reaction and are considered to provide cooked flavor [16,34]. Among seven pyrazines, the relative abundance of 2,5-dimethyl pyrazine dominated. This compound is responsible for burning aroma [16].

Terpenes and terpenoid structures have been detected as secondary metabolites in plants, animals, and microorganisms. Some of them include highly odor-active compounds. Among the terpenes identified, 3-carene and D-limonene are the most relevant. The odor of 3-carene is associated with resinous, coniferous forest odor [82]. D-limonene is one of the most common terpenes in nature, occurring in citrus and a wide variety of other plant species, and has a pleasing orange scent [83]. Small amounts of this terpene were previously detected in TM edible oil.

Summing up, the volatile profile of analyzed TM oil samples was characterized mainly by the distinguished abundance of aroma-dependent hexanal and other aliphatic aldehydes resulting from the oxidation of PUFAs. The second important volatile compounds were Strecker aldehydes and pyrazines, which may provide a wide range of aromas (roasted, fatty, toasty, and roasted). Some volatiles have not been associated with aroma attributes such as hydrocarbons; they may be important as an indicator of the quality of TM oil.

### 3.5. Physical Properties

The viscosity and surface tension analysis results of the TM oil are shown in Table 5. As expected, no significant differences were observed in either parameter between batches. However, viscosity values are significantly lower, 5 times less, than those previously reported in *Tenebrio molitor* oil [37,38] but similar to some edible vegetable oils such as corn, soy, or sunflower [84,85]. These differences could be attributed to the shear rate applied and the filtration step during the obtaining procedure.

**Figure 2:**
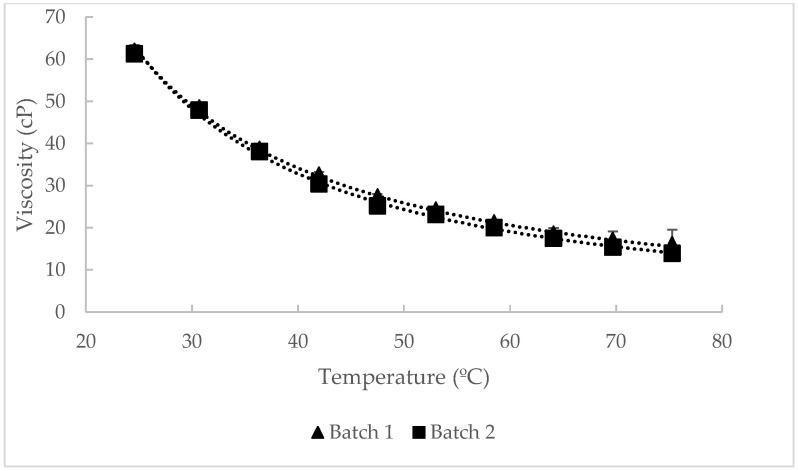
Viscosity curve at different temperatures of *Tenebrio molitor* oil.

As expected, TM oil samples showed a Newtonian behavior at different temperatures (Figure 2). This behavior was also observed in different vegetable edible oils [86] and those obtained from insects [38,41]. Surface tension results were also similar to some cold-pressed edible oils like flaxseed, avocado, or EVOO [85].

Insect oil for human consumption is studied little for application in the food industry. It is mainly used in cosmetics and pharmaceutical products [12]. However, from a practical point of view, knowledge about physical properties such as viscosity or surface tension of TM oil is necessary for assessing their behavior in water/oil food emulsions, oleo gels, or even as a frying medium [87,88,89,90,91].

### 3.6. Aroma Sensory Analysis

**Figure 3:**
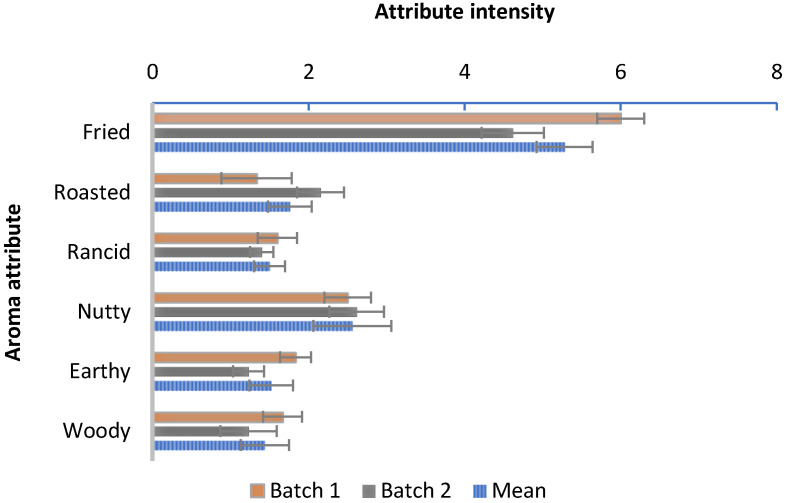
Results of the sensory estimation of aroma attributes of the *Tenebrio molitor* oil (n = 13) mean ± SD. Attribute intensity scale: 0—very faint; 2—slightly; 4—moderate; 6—intense; 8—very intense.

The results of the sensory estimation of aroma attributes of TM oil are shown in Figure 3. The panelists found a predominant odor of fried products in TM oil. This odor could be related to the abundance of volatile compounds derived from lipid oxidation, such as linear aldehydes, specifically hexanal, heptanal, and carboxylic acids (Table 4).

The intensity of nutty and roasted aromas was assayed as more than slightly. This is concordant with the results of instrumental analysis of the volatile profile conducted by GC/MS of TM oil, in which a substantial abundance of Strecker aldehydes and pyrazines were assayed [15] (Table 4). As previously indicated, the nutty and roasted odor originates from compounds generated during the Maillard reaction in the larvae drying [37]. Pyrazines, especially 2,5-dimethyl pyrazine, are associated with a nutty odor but also contribute to the formation of woody and roasted aromas [92]. The detection of earthy odors could be related to ketones, such as 3-octen-2-one, and alcohols like 1-octen-3-ol; whereas the woody odor is also supported by the presence of terpenes: α and β-pinene and 3-carene. Despite the low content, their odor strength is medium–high [92]. During the sensory estimation of the aroma profile, a low-intensity rancid aroma was assayed, which was scored by the sensory panel as slightly intensive. A relatively high abundance of hexanal, nonanal, pentanal, and carboxylic acids (acetic and hexanoic acid) detected in the volatile profile of TM oil probably contributed to the formation of n rancid aroma (Table 4).

## 4. Conclusions and Future Perspectives

It can be concluded that the by-product *Tenebrio molitor* oil obtained during defatted mealworm powder production presents interesting characteristics for its potential use as a nutraceutical ingredient in food formulations.

*Tenebrio molitor* oil may represent a novel dietary source of valuable essential PUFAs: linoleic acid—C18:2 *9c12c* and α-linolenic acid—C18:3 *9c12c15c* and its containment of tocopherols and carotenoids distinguished it which are an effective lipophilic antioxidant capable of scavenging lipid peroxyl radicals. Results of instrumental assays revealed this oil as a source of phenolic compounds. In the analyzed first-time phenolic profile of *Tenebrio molitor* oil, a noticeable amount of apigenin was noted among nine detected phenolic compounds. The substantial presence of lipophilic and phenolic compounds contributed to their antioxidative potential.

Sensory estimation revealed the dominance of fried and nutty aromas, probably because of the substantial abundance of Strecker aldehydes and pyrazines in its volatile profile. Among volatiles, the distinguished abundance of aroma-dependent aliphatic aldehydes, mainly hexanal, resulting from the oxidation of PUFAs should be noted. The results indicated that the technological process needs modification to limit the formation of lipid oxidation products, especially by applying lower temperatures and sustainable solvents.

This preliminary study on the composition and properties of *Tenebrio molitor* oil encourages its use as an ingredient for food, pharmaceutical, and cosmetics purposes. However, future studies are needed to analyze potential contaminants and improve the sensory attributes of oil, i.e., by deodorization. Also, animal studies are required to elaborate on the long-term health effects of consuming *Tenebrio molitor* oil.

## Figures and Tables

**Table 1:** Indicators of oxidative and hydrolytic stability of *Tenebrio molitor* oil samples (n = 6).

	Batch 1	Batch 2	Mean ± SD
Free fatty acid—FFA (%)	1.13 ± 0.00 ^a^	1.13 ± 0.00 ^a^	1.13 ± 0.00
Peroxide value—PV (mEq O active/kg oil)	13.37 ± 0.04 ^a^	11.65 ± 0.02 ^b^	12.51 ± 0.90
Conjugated diene—CD (μmol/g)	2.67 ± 0.02 ^a^	2.66 ± 0.04 ^a^	2.65 ± 0.05
Conjugated triene—CT (μmol/g)	0.70 ± 0.01 ^a^	0.73 ± 0.01 ^a^	0.71 ± 0.01
Induction time (h)	2.75 ± 0.14 ^a^	2.75 ± 0.16 ^a^	2.75 ± 0.16

Different letters in each row indicate significant differences (*p* < 0.05) between batches.

**Table 2:** Fatty acids composition (g/100 g FA), nutritional quality indexes, and cholesterol of *Tenebrio molitor* oil (n = 6).

	Batch 1	Batch 2	Mean ± SD
C8:0	N.D	N.D	N.D
C10:0	0.02 ± 0.00 ^a^	0.02 ± 0.00 ^a^	0.02 ± 0.00
C12:0	0.36 ± 0.00 ^a^	0.31 ± 0.00 ^b^	0.33 ± 0.03
C13:0	0.06 ± 0.00 ^a^	0.06 ± 0.00 ^a^	0.06 ± 0.00
C14:0	2.87 ± 0.01 ^a^	2.64 ± 0.01 ^b^	2.76 ± 0.12
C14:1	0.02 ± 0.00 ^a^	0.03 ± 0.00 ^a^	0.02 ± 0.00
C15:0	0.16 ± 0.00 ^a^	0.19 ± 0.00 ^a^	0.18 ± 0.01
i-C16:0	0.01 ± 0.00 ^a^	0.01 ± 0.00 ^a^	0.01 ± 0.00
C16:0	19.40 ± 0.02 ^a^	19.14 ± 0.03 ^b^	19.27 ±0.14
C16:1*7c*	0.61 ± 0.00 ^a^	0.52 ± 0.01 ^a^	0.56 ± 0.04
C16:1*9c*	1.79 ± 0.00 ^a^	1.60 ± 0.00 ^b^	1.70 ± 0.11
a-C17:0	0.01 ± 0.01 ^a^	0.02 ± 0.00 ^a^	0.02 ± 0.00
C17:0	0.12 ± 0.00 ^a^	0.14 ± 0.01 ^a^	0.13 ± 0.02
i-C18:0	0.09 ± 0.00 ^a^	0.10 ± 0.00 ^a^	0.10 ± 0.01
C18:0	2.72 ± 0.03 ^a^	3.05 ± 0.05 ^b^	2.89 ± 0.18
C18:1*9c*	38.30 ± 0.05 ^a^	35.31 ± 0.24 ^b^	36.81 ± 1.64
C18:1*11c*	1.10 ± 0.02 ^a^	1.06 ± 0.05 ^a^	1.08 ± 0.04
C18:2*9c12c* (ɷ-6)	30.84 ± 0.01 ^a^	34.04 ± 0.13 ^b^	32.44 ± 1.76
C18:3*9c12c15c* (ɷ-3)	1.35 ± 0.01 ^a^	1.56 ± 0.02 ^b^	1.46 ± 0.12
C20:0	0.06 ± 0.00 ^a^	0.06 ± 0.00 ^a^	0.06 ± 0.00
C20:1	0.08 ± 0.00 ^a^	0.09 ± 0.00 ^a^	0.08 ± 0.01
C20:2 (ɷ-6)	0.04 ± 0.00 ^a^	0.05 ± 0.00 ^a^	0.04 ± 0.00
ƩSFA	25.87 ± 0.03 ^a^	25.74 ± 0.06 ^a^	25.81 ± 0.08
ƩMUFA	41.90 ± 0.03 ^a^	38.61 ± 0.20 ^b^	40.26 ± 1.81
ƩPUFA	32.23 ± 0.02 ^a^	35.65 ± 0.15 ^b^	33.94 ± 1.88
SFA/UFA	0.35 ± 0.00 ^a^	0.35 ± 0.00 ^a^	0.35 ± 0.00
PUFA/SFA	1.25 ± 0.00 ^a^	1.39 ± 0.00 ^b^	1.32 ± 0.08
ɷ-6/ɷ-3 ratio	22:1 ^a^	23:1 ^b^	22:1
COX	3.89 ± 0.00 ^a^	4.23 ± 0.02 ^a^	4.06 ± 0.19
Cholesterol (mg/100 g)	146 ± 7.40 ^a^	147 ± 5.09 ^a^	146 ± 5.75
Nutritional Quality Indexes			
IT	0.62 ± 0.00 ^a^	0.61 ± 0.00 ^a^	0.61 ± 0.01
AI	0.42 ± 0.00 ^a^	0.40 ± 0.00 ^a^	0.41 ± 0.01
HH	3.71 ± 0.78 ^a^	3.51 ± 0.55 ^a^	3.51 ± 0.55
HPI	2.37 ± 0.00 ^a^	2.47 ± 0.01 ^a^	2.42 ± 0.06

Different letters in each row indicate significant differences (*p* < 0.05) between batches. SFA: saturated fatty acids; MUFAs: monounsaturated fatty acids; PUFAs: polyunsaturated fatty acids; UFAs: unsaturated fatty acids; COX: calculated oxidizability value; IT: thrombogenicity index; AI: atherogenicity index; HH: hypocholesterolemic–hypercholesterolemic index; HPI: health-promoting index.

**Table 3:** Bioactive compounds and antioxidative potential of *Tenebrio molitor* oil (n = 6).

	Batch 1	Batch 2	Mean ± SD
Total carotenoids (mg/kg oil)	14.19 ± 0.76 ^a^	12.29 ± 0.16 ^b^	13.65 ± 1.60
Total phenolic compounds—TPC (mg GAE/kg oil)	76.37 ± 4.54 ^a^	71.82 ± 3.71 ^b^	74.09 ± 4.47
Apigenin (mg/100 g oil)	1.36 ± 0.29 ^a^	0.53 ± 0.04 ^b^	0.94 ± 0.51
Total tocopherol (mg/kg oil)	120.8 ± 3.10 ^a^	90.8 ± 2.80 ^b^	105.8 ± 4.60
α-tocopherol	66.9 ± 3.90 ^a^	44.6 ± 2.00 ^b^	55.8 ± 3.25
γ-tocopherol	50.3 ± 4.5 ^a^	43.3 ± 3.0 ^a^	46.8 ± 3.95
δ-tocopherol	3.6 ± 0.4 ^a^	2.8 ± 0.2 ^b^	3.2 ± 0.5
*Antioxidant capacity*			
DPPH			
mM TE/kg oil	2.40 ± 0.32 ^a^	1.66 ± 0.22 ^b^	2.03 ± 0.47
% Inhibition	48.34 ± 6.05 ^a^	34.51 ± 4.14 ^b^	41.42 ± 8.88
ABTS			
mM TE/kg oil	1.71 ± 0.18 ^a^	1.62 ± 0.07 ^a^	1.66 ± 0.13
% Inhibition	36.56 ± 4.11 ^a^	34.68 ± 1.74 ^a^	35.62 ± 3.00

Different letters in each row indicate significant differences (*p* < 0.05) between batches. GAE: gallic acid equivalent; TE: Trolox equivalent.

**Table 4:** Relative content (%) of volatile compounds in *Tenebrio molitor* oil (n = 6).

	Batch 1	Batch 2	Mean ± SD
***Aliphatic Hydrocarbons***			
Pentane	1.96 ± 0.22 ^a^	1.40 ± 0.08 ^b^	1.68 ± 0.36
Decane	0.74 ± 0.06 ^a^	1.26 ± 0.06 ^b^	1.00 ± 0.29
(4e)-4-undecene	0.15 ± 0.03 ^a^	0.36 ± 0.07 ^b^	0.25 ± 0.13
(5z)-5-undecene	0.30 ± 0.07 ^a^	0.21 ± 0.03 ^a^	0.25 ± 0.08
Undecane	0.20 ± 0.02 ^a^	0.57 ± 0.01 ^b^	0.38 ± 0.21
3-methylundecane	0.11 ± 0.02 ^a^	0.12 ± 0.01 ^a^	0.11 ± 0.02
Dodecane	0.25 ± 0.05 ^a^	0.54 ± 0.05 ^b^	0.39 ± 0.17
Tridecane	0.13 ± 0.01 ^a^	0.23 ± 0.02 ^b^	0.18 ± 0.06
Tetradecane	0.07 ± 0.03 ^a^	0.27 ± 0.05 ^b^	0.17 ± 0.12
***Total***	3.90 ± 0.24 ^a^	4.95 ± 0.21 ^b^	4.42 ± 0.62
***Aromatic hydrocarbons***			
Toluene	0.35 ± 0.04 ^a^	1.25 ± 0.17 ^b^	0.80 ± 0.51
Ethylbenzene	0.26 ± 0.02 ^a^	0.44 ± 0.01 ^b^	0.35 ± 0.10
p-Xylene (1,4-dimethylbenzene)	0.47 ± 0.04 ^a^	0.75 ± 0.03 ^b^	0.61 ± 0.16
Styrene (ethenylbenzene)	0.33 ± 0.02 ^a^	0.27 ± 0.02 ^a^	0.30 ± 0.04
O-xylene (1,2-dimethylbenzene)	0.31 ± 0.02 ^a^	0.32 ± 0.00 ^a^	0.32 ± 0.02
Propylbenzene	0.22 ± 0.01 ^a^	0.24 ± 0.01 ^a^	0.23 ± 0.02
1-Ethyl-4-methylbenzene (toluene, p-ethyl)	2.15 ± 0.19 ^a^	1.85 ± 0.32 ^a^	2.00 ± 0.33
1,3,5-Trimethylbenzene	0.63 ± 0.04 ^a^	0.85 ± 0.02 ^b^	0.74 ± 0.12
1,2,4-Trimethylbenzene	2.54 ± 0.21 ^a^	3.50 ± 0.50 ^b^	3.02 ± 0.67
1,2,3-Trimethylbenzene	1.63 ± 0.30 ^a^	2.52 ± 0.37 ^a^	2.07 ± 0.61
1,3-Diethylbenzene	0.18 ± 0.02 ^a^	0.25 ± 0.02 ^b^	0.21 ± 0.04
1-Methyl-3-propylbenzene (Toluene, m-propyl)	0.41 ± 0.04 ^a^	0.57 ± 0.05 ^b^	0.49 ± 0.10
1,4-Diethylbenzene	1.13 ± 0.19 ^a^	0.78 ± 0.06 ^b^	0.96 ± 0.24
1-Ethyl-3,5-dimethylbenzene)	0.36 ± 0.04 ^a^	0.57 ± 0.05 ^b^	0.47 ± 0.12
1-Methyl-2-propylbenzene (Toluene, o-propyl)	0.77 ± 0.05 ^a^	0.48 ± 0.05 ^b^	0.62 ± 0.17
2-Ethyl-1,3-dimethylbenzene (m-Xylene, 2-ethyl-)	0.87 ± 0.08 ^a^	0.66 ± 0.07 ^b^	0.77 ± 0.14
4-Ethyl-1,2-dimethylbenzene (o-Xylene, 4-ethyl-)	0.37 ± 0.03 ^a^	0.62 ± 0.05 ^b^	0.50 ± 0.15
1,2,4,5-tetramethylbenzene	0.56 ± 0.06 ^a^	0.37 ± 0.10 ^b^	0.47 ± 0.14
***Total***	13.53 ± 0.48 ^a^	16.30 ± 1.17 ^b^	14.91 ± 1.80
***Alcohols***			
1-Pentanol	1.71 ± 0.26 ^a^	2.96 ± 0.27 ^b^	2.34 ± 0.74
1-Octen-3-ol	0.43 ± 0.04 ^a^	0.59 ± 0.03 ^b^	0.51 ± 0.09
1,5-Heptadiene-3,4-diol	2.92 ± 0.52 ^a^	1.76 ± 0.01 ^b^	2.34 ± 0.75
***Total***	5.06 ± 0.28 ^a^	5.30 ± 0.25 ^a^	5.18 ± 0.32
***Aldehydes***			
2-Methylpropanal	1.25 ± 0.07 ^a^	0.90 ± 0.07 ^b^	1.08 ± 0.21
3-Methylbutanal	0.90 ± 0.09 ^a^	1.02 ± 0.13 ^a^	0.96 ± 0.14
2-Methylbutanal	0.88 ± 0.13 ^a^	1.49 ± 0.08 ^b^	1.18 ± 0.35
Pentanal	4.58 ± 0.57 ^a^	4.74 ± 0.38 ^a^	4.66 ± 0.54
Hexanal	40.66 ± 2.12 ^a^	36.04 ± 1.91 ^a^	38.35 ± 3.36
2-Hexenal	0.15 ± 0.02 ^a^	0.19 ± 0.01 ^b^	0.17 ± 0.03
Heptanal	1.32 ± 0.08 ^a^	1.46 ± 0.05 ^a^	1.39 ± 0.11
Benzaldehyde	0.42 ± 0.03 ^a^	0.50 ± 0.05 ^a^	0.46 ± 0.06
Octanal	2.38 ± 0.14 ^a^	2.96 ± 0.19 ^b^	2.67 ± 0.37
(E)-2-Octenal	0.46 ± 0.05 ^a^	0.34 ± 0.03 ^b^	0.40 ± 0.08
Nonanal	2.56 ± 0.37 ^a^	2.55 ± 0.24 ^a^	2.56 ± 0.35
Decanal	0.19 ± 0.04 ^a^	0.27 ± 0.02 ^b^	0.23 ± 0.06
2-Butyl-2-octenal	0.26 ± 0.10 ^a^	0.24 ± 0.05 ^a^	0.25 ± 0.09
***Total***	56.02 ± 2.11 ^a^	52.70 ± 1.98 ^a^	54.36 ± 2.89
***Carboxylic acids***			
Acetic acid	1.33 ± 0.17 ^a^	0.90 ± 0.11 ^b^	1.12 ± 0.29
3-Methylbutanoic acid	0.21 ± 0.04 ^a^	0.27 ± 0.02 ^a^	0.24 ± 0.05
Hexanoic acid	5.36 ± 1.24 ^a^	2.45 ± 0.52 ^b^	3.91 ± 1.90
***Total***	6.91 ± 1.18 ^a^	3.63 ± 0.61 ^b^	5.27 ± 2.07
***Ketones***			
2-Butanone	0.94 ± 0.07 ^a^	0.60 ± 0.05 ^b^	0.77 ± 0.20
2-Heptanone	1.34 ± 0.18 ^a^	1.24 ± 0.07 ^a^	1.29 ± 0.16
2,3-Octanedione	0.13 ± 0.01 ^a^	0.37 ± 0.29 ^b^	0.25 ± 0.26
3-Octen-2-one	1.46 ± 0.14 ^a^	1.06 ± 0.17 ^b^	1.26 ± 0.28
3,5-Octadien-2-one	0.35 ± 0.03 ^a^	0.27 ± 0.06 ^a^	0.31 ± 0.07
2-Decanone	0.20 ± 0.05 ^a^	0.24 ± 0.04 ^a^	0.22 ± 0.05
***Total***	4.43 ± 0.23 ^a^	3.80 ± 0.42 ^b^	4.11 ± 0.51
***Pyrazines***			
Methyl pyrazine	0.31 ± 0.03 ^a^	0.51 ± 0.02 ^b^	0.41 ± 0.11
2,5-Dimethyl pyrazine	1.17 ± 0.12 ^a^	1.73 ± 0.26 ^b^	1.45 ± 0.37
2,6-Dimethyl pyrazine	0.15 ± 0.03 ^a^	0.34 ± 0.02 ^b^	0.25 ± 0.11
2-Ethyl-5-methyl pyrazine	0.61 ± 0.04 ^a^	0.66 ± 0.04 ^a^	0.63 ± 0.05
3-Ethyl-2,5-dimethyl pyrazine	0.68 ± 0.07 ^a^	0.51 ± 0.05 ^a^	0.60 ± 0.11
5-Ethyl-2,3-dimethyl pyrazine	0.74 ± 0.04 ^a^	0.55 ± 0.06 ^b^	0.65 ± 0.12
3,5-Diethyl-2-methyl pyrazine	0.07 ± 0.01 ^a^	0.06 ± 0.01 ^a^	0.06 ± 0.01
***Total***	3.74 ± 0.27 ^a^	4.36 ± 0.07 ^b^	4.05 ± 0.48
***Terpenes***			
α-Pinene	0.17 ± 0.02 ^a^	0.55 ± 0.02 ^b^	0.36 ± 0.21
β-Pinene	0.18 ± 0.02 ^a^	0.31 ± 0.01 ^b^	0.25 ± 0.07
3-Carene	1.93 ± 0.10 ^a^	2.27 ± 0.13 ^b^	2.10 ± 0.23
D-Limonene	1.89 ± 0.44 ^a^	3.32 ± 0.67 ^b^	2.61 ± 1.00
α-Copaene	0.08 ± 0.01 ^a^	0.16 ± 0.01 ^b^	0.12 ± 0.05
***Total***	4.25 ± 0.42 ^a^	6.60 ± 0.55 ^b^	5.43 ± 1.40
***Others***			
2-Pentylfuran	1.22 ± 0.08 ^a^	1.43 ± 0.08 ^b^	1.32 ± 0.14
Ethyl acetate	0.53 ± 0.07 ^a^	0.52 ± 0.06 ^b^	0.53 ± 0.07
1H-Pyrrole	0.17 ± 0.03 ^a^	0.17 ± 0.02 ^a^	0.17 ± 0.03
1H-Pyrrole, 1-butyl-	0.25 ± 0.01 ^a^	0.25 ± 0.02 ^a^	0.25 ± 0.02

Different letters in each row indicate significant differences (*p* < 0.05) between batches.

**Table 5:** Physical properties of the *Tenebrio molitor* oil (n = 6).

	Batch 1	Batch 2	Mean ± SD
Viscosity (cP) at 25 °C	61.47 ± 0.58 ^a^	61.90 ± 0.95 ^a^	61.68 ± 0.74
Surface tension (mN/m)	36.50 ± 0.87 ^b^	36.00 ± 1.00 ^b^	36.25 ± 0.88

Different letters in each row indicate significant differences (*p* < 0.05) between batches.

## Data Availability

The original contributions presented in this study are included in the article and Supplementary Material. Further inquiries can be directed to the corresponding author.

## Supplementary Materials

The following supporting information can be downloaded at: https://www.mdpi.com/article/10.3390/foods13233867/s1, Table S1. Content of specific phenolic compounds in *Tenebrio molitor* oil (mg/100 g oil).
