# Supplementary material for: Exploring the Potential of Yellow Mealworm (Tenebrio molitor) Oil as a Nutraceutical Ingredient
Source: Foods. 2024 Nov 29;13(23):3867. doi: 10.3390/foods13233867 (PMC11640693; doi:10.3390/foods13233867)
Supplement: Supplementary file 1 [file foods-13-03867-s001.zip › Suplementary materials Table S1.pdf]

Table S1. Content of specific phenolic compounds in *Tenebrio molitor* oil (mg/100g oil)

**Phenolic compound**	**Batch 1**	**Batch 2**	**Mean**
Malvidin	< LOQ	< LOQ	-
Chlorogenic acid	< LOQ	< LOQ	-
Apigenin	1.36 ± 0.29^{a}	0.53 ± 0.04^{b}	0.94 ± 0.51
Epicatechin	< LOQ	< LOQ	-
Naringenin	< LOQ	< LOQ	-
Quercetin	< LOQ	< LOQ	-
Gallic acid	< LOQ	< LOQ	-
Ferulic acid	< LOQ	< LOQ	-
Coumaric acid	< LOQ	< LOQ	-

Mean values ± standard deviations. Different letters in each row indicate significant differences ($p<0.05$) between batches. LOQ= 0.10mg/L
